# Oral Squamous Cell Carcinomas Developing from Oral Lichen Planus: A 5–21 year Retrospective Study

**DOI:** 10.1007/s12663-022-01729-y

**Published:** 2022-05-28

**Authors:** Kawe Sagheb, Sebastian Blatt, Roman-Kia Rahimi-Nedjat, Abdullatif Lingawi, Eik Schiegnitz, Vinay V. Kumar, Christian Walter, Keyvan Sagheb

**Affiliations:** 1 Department of Oral and Maxillofacial Surgery – Plastic Surgery, University Medical Center of the Johannes Gutenberg-University Mainz, Augustusplatz 2, 55131 Mainz, Germany; 2 Department of Prosthetic Dentistry, University Medical Center of the Johannes Gutenberg-University Mainz, Augustusplatz 2, 55131 Mainz, Germany; 3 Clinic for Psychiatry, Psychotherapy and Psychosomatic, St. Valentinus Hospital, Suttonstraße 24, 65399 Kiedrich, Germany; 4 Private Practice for Oral- and Maxillofacial Surgery - Facial Plastic Surgery, Mediplus Clinic, Haifa-Allee 20, 55128 Mainz, Germany; 5 Department of Oral and Maxillofacial Surgery – Integrated Head and Neck Surgery, Sri Shankara Cancer Hospital and Research Center, Basavangudi, Bangalore, 560004 India; 6 Department of Plastic and Maxillofacial Surgery, Uppsala University Hospital, Uppsala, Sweden

**Keywords:** Oral cancer, Head and neck cancer, Oral lichen planus, Lymphatic metastasis, Relapse

## Abstract

**Background and Aims:**

There is insufficient data regarding clinical characteristics, relapse rates, as well as lymph node metastasis of squamous cell carcinomas of the oral cavity (OSCC) developing from oral lichen planus (OLP-OSCC). The aim of this retrospective study was to evaluate clinical characteristics, as well as relapse, recurrence and survival rates of OLP-OSCC.

**Methods:**

In a retrospective monocenter analysis, all consecutive patients with an OSCC treated in the time period 1st January 2000–December 31 2016 were reviewed. All patients with OSCC developing from OLP/OLL (oral lichenoid lesions) were identified and analyzed for epidemiological data, risk profile, location of primary tumor, pTNM classification, lymph node metastasis, primary therapy, recurrence, and outcome.

**Results:**

A total of 103 patients (45%♂/ 55%♀) with an average age of 62 ± 14 year were included in this study. At the time of initial diagnosis, 17% (*n* = 18) of patients had cervical metastases (CM) whereas only 11% (11 patients) displayed advanced tumor sizes (*T* > 2). *T*-status (*p* = 0.003) and histopathological grading (*p* = 0.001) had an impact on the incidence of CM. 39.6% of the patients developed a relapse after an average of 24 months with a mean of two recurrences per patient. Advanced tumor size had a significant impact on the 5 year overall survival and was associated with disease-free survival of the patients (*p* < 0.001, respectively *p *= 0.004).

**Conclusion:**

Although initial lymph node metastases were not more frequent, more aggressive recurrence patterns compared to OSCC were seen for OLP-OSCC. Therefore, based on the study results, a modified recall for these patients is suggested.

## Introduction

Oral cancer is one of the most common malignancies worldwide, with an annual incident rate of 4.0 per 100,000 and a worldwide mortality rate of 2.7 per 100,000 [1]. Apart from the classic risk factors like smoking and alcohol, potentially malignant disorders are the most common predisposing factors for the development of oral squamous cell carcinoma (OSCC). Oral lichen planus (OLP), according to the World Health Organization defined as “a chronic inflammatory disease of the skin and the oral mucosa of unknown etiology”, is considered to be one of the most common and therefore most relevant premalignant conditions for the development of OSCC with inconsistent described transformation rates of up to 1–3% [2–4].

Although the exact etiopathogenesis of the OLP has not been inconclusively defined, the immunological system is believed to play a leading role for this chronic inflammatory disease that frequently involves the oral mucosa [5, 6]. OLP has a higher prevalence in women, commonly developing on the buccal mucosa followed by the tongue [5, 6]. Clinically, OLP is present in six different forms: reticular, papular, bullous, plaque, atrophic, and erosive forms, the former being the most common and the latter having a higher chance of malignant transformation [2]

Recent molecular studies support the hypothesis of OLP as a preneoplastic inflammatory model with an inflammatory cytokine-rich microenvironment favoring tumor promotion [6, 7]. The erythematous and erosive form of OLP is especially related to the development of OSCC [6]. The first case of malignant transformation of an OLP was described at the beginning of the twentieth century, followed by a large number of case reports [5]. Since then several reviews emphasized on existing follow-up studies. Due to the low proportion of OSCC developing from OLP (OLP-OSCC) of the oral cavity, there is little isolated data regarding the frequency of lymph node metastasis and clinical characteristics such as relapse and outcome [8–10]. Compounding these issues, are the lack of generally accepted diagnostic criteria and the heterogeneity of follow-up studies based only on clinical diagnostic criteria or the combination of clinical and histological criteria [6]. In addition, the histologic diagnosis of OLP is challenging with moderate inter- and intraobserver variability [11] as well as the difficulty to discriminate from other oral lichenoid lesions (OLL) [12]. Treatment of OLP-OSCC remains challenging: chronic inflammation of the oral mucosa often leads to cervical lymphadenitis that complicates neck management, and high recurrence rates hamper first line therapy options and worsen patient outcomes.

The aim of this retrospective study was therefore to analyze the subgroup OLP-OSCC with special regards on clinical features, recurrence patterns and cervical metastasis.

## Patients and Methods

In this retrospective monocenter analysis, all consecutive patients with OLP-OSCC treated between January 1st 2000 and December 31st 2016 in the Department of Oral and Maxillofacial Surgery – Plastic Surgery of the University Medical Center Mainz, Germany, were analyzed.

For inclusion criteria, medical records of all OSCC patients with a histologically confirmed OLP prior to tumor development were reviewed. Patients with distant metastasis at the time of primary presentation, OSCC without OLP, as well as malignancies that were not treatment-naïve were excluded from this analysis. The data entry was accomplished using conventional and electronic patient records. Epidemiological data, risk profile for nicotine and alcohol, location of primary tumor, pTNM, lymph node metastasis, primary therapy, recurrence and outcome were analyzed. The 7th version of the TNM-classification by the Union for International Cancer Control (UICC) was applied due to the retrospective manner of this study. All patients were treated surgically in accordance with current guideline recommendations after interdisciplinary tumor board advice. A safe resection margin was histologically defined > 5 mm (*R*0). Recurrence was defined as either local carcinoma recurrence at the same anatomic site within 5 years after primary treatment or regional recurrence meaning lymph node metastases within 5 years after primary treatment. A tumor recurrence after 5 years was defined as a second cancer.

The study was conducted within the Helsinki Declaration of Human Rights and in accordance with the guidelines of the local ethics committee (Ethikkommission Ärztekammer Rheinland-Pfalz, Mainz, Germany). According to the hospital law of Rhineland-Palatine, Germany (*Krankenhauslandesgesetz),* no specific approval by the local ethics committee is necessary for a retrospective data study.

SPSS 23.0 for Windows (IBM Deutschland GmbH, Ehningen, Germany) was used for statistical analysis. For multivariate analysis, ANOVA testing was performed to detect possible risk factors for tumor relapse and overall survival. For graphic display, Kaplan–Meier plots in combination with log-rank Mantel–Cox regression were used. Due to multiple testing, a Bonferoni correction was applied and a *p* value ≤ 0.0025 was defined as statistically significant.

## Results

### Epidemiological Data

A total of 103 patients with a mean age of 62 ± 14 year were included in the study (Table 1). 55% (*n* = 57, 64 ± 14 year) were female and 45% (*n* = 46, 59 ± 14 year) male. 65% of the patients consumed alcohol and/or cigarettes regularly, and 71% had one or more underlying diseases apart from OSCC. On an average, women had more underlying diseases (3 ± 2) than men (2 ± 1). The most frequent conditions were cardiovascular diseases and diabetes mellitus.

**Table 1:** Epidemiological data and TNM classification of the patients with OLP-OSCC at the point of initial diagnosis

	Female	Male	All patients
Gender	55% (*n* = 57)	45% (*n* = 46)	(*n* = 103)
*Risk profile*			
Positive	61% (*n* = 35)	70% (*n* = 32)	65% (*n* = 67)
Negative	39% (*n* = 22)	30% (*n* = 14)	35% (*n* = 36)
*Underlying diseases*			
Yes	70% (*n* = 40)	72% (*n* = 33)	71% (*n* = 73)
No	30% (*n* = 17)	28% (*n* = 13)	30% (*n* = 30)
Age (years ± SD)	64 ± 14	59 ± 14	62 ± 14
*T-stage*			
*T*1	60% (*n* = 34)	61% (*n* = 28)	60% (*n* = 62)
*T*2	31% (*n* = 18)	26% (*n* = 12)	29% (*n* = 30)
*T*3	2% (*n* = 1)	% (*n* = 0)	1% (*n* = 1)
*T*4	7% (*n* = 4)	13% (*n* = 6)	10% (*n* = 10)
*N*-stage			
*N*0	84% (*n* = 48)	80% (*n* = 37)	83% (*n* = 85)
*N* +	16% (*n* = 9)	20% (*n* = 9)	17% (*n* = 18)
*G-stage*			
*G*1	51% (*n* = 29)	37% (*n* = 17)	45% (*n* = 46)
*G*2	49% (*n* = 28)	57% (*n* = 26)	52% (*n* = 54)
*G*3	0% (*n* = 0)	6% (*n* = 3)	3% (*n* = 3)
*Tumor-stage*			
I	60% (*n* = 34)	59% (*n* = 27)	59% (*n* = 61)
II	23% (*n* = 13)	15% (*n* = 7)	19% (*n* = 20)
III	2% (*n* = 1)	9% (*n* = 4)	5% (*n* = 5)
IV	16% (*n* = 9)	17% (*n* = 8)	17% (*n* = 17)

### Localization and pTNM-Classification

Most often the cancer was localized at the margin of the tongue (28%), followed by the buccal mucosa (25%) and the lower jaw (22%). Rare localizations were the upper jaw (14%) and the floor of the mouth (11%). In majority, 89% (*n* = 92) of the tumors were detected at an early stage (*T*1 or *T*2), eleven patients showed larger tumor sizes *T*3 or *T*4 (11%). 79% (*n* = 81) were summarized as stage I/II, 21% of the cases (*n* = 22) were staged as III/IV in accordance with the 7th UICC version (Table 1). Histologically, 56% (*n* = 57) of the resected specimen displayed “moderate to poorly differentiated” grading. For the majority of the patients (88%, *n* = 93), a sufficient adequate safety margin (*R* = 0) was achieved by primary surgical therapy (Table 1). Eighteen patients (17%) had developed cervical metastasis at the time of initial diagnosis which was significantly associated with an advanced tumor size (*T*3–4; *p* = 0.003). Furthermore, a higher grading showed an influence on occurrence of cervical metastasis (*G*2–3; *p* = 0.001), however, neither gender, age, risk factors, underlying diseases, nor anatomic site of tumor manifestation (Table 2) showed a relation to cervical metastasis. An estimation of the percentage of occult metastases was not possible since 96% (*n* = 102) of patients had an abnormal cervical lymph node status in CT and sonography, preoperatively.

**Table 2:** Presence of CM according to different clinical parameters at the point of initial diagnosis

		*N*0	*N* +	Portion of *N* + to the respective factor %	
*T*1	*n* = 62	*n* = 61	*n* = 1	2	*p* = 0.003*
*T*3	*n* = 30	*n* = 19	*n* = 11	37	
*T*3	*n* = 1	*n* = 0	*n* = 1	100	
*T*4	*n* = 10	*n* = 5	*n* = 5	50	
*G*1	*n* = 46	*n* = 45	*n* = 1	2	*p* = 0.001
*G*2	*n* = 54	*n* = 38	*n* = 16	30	
G3	*n* = 3	*n* = 2	*n* = 1	33	
Male	*n* = 46	*n* = 37	*n* = 9	19	*p* = 0.729
Female	*n* = 57	*n* = 50	*n* = 7	15	
Positive RF	*n* = 67	*n* = 56	*n* = 11	16	*p* = 0.544
Negative RF	*n* = 36	*n* = 29	*n* = 7	19	
Positive UD	*n* = 73	*n* = 62	*n* = 11	15	
Negative UD	*n* = 30	*n* = 23	*n* = 7	23	*p* = 0.456
Upper jaw	*n* = 15	12	3	20	*p* = 0.527
Buccal mucosa	*n* = 25	24	1	4	
Tongue	*n* = 28	25	3	10	
Lower jaw	*n* = 23	13	10	44	
Mouth floor	*n* = 12	11	1	8	
Age ≤ 50 year	*n* = 22	21	1	5	*p* = 0.073
Age > 50 year	*n* = 81	67	14	17	

*statistically significant

### Primary Therapy

For primary therapy, all patients were treated surgically in accordance with then current guidelines and following the recommendations of the interdisciplinary tumor board. For reconstruction of the defect, in 46% (*n* = 49), primary wound closure was sufficient, 40% (*n* = 42) were reconstructed with a local and 13% (*n* = 14) with a microvascular anastomosed flap. 24.5% of the patients (*n* = 26) needed adjuvant radiation. Thirteen cases needed adjuvant radiation in addition to cisplatin chemotherapy as a radiosensitizer, because of extended *T*-status and multiple lymph node metastases with or without extracapsular spread.

### Follow-Up and Patient’s Outcome

**Fig. 1:**
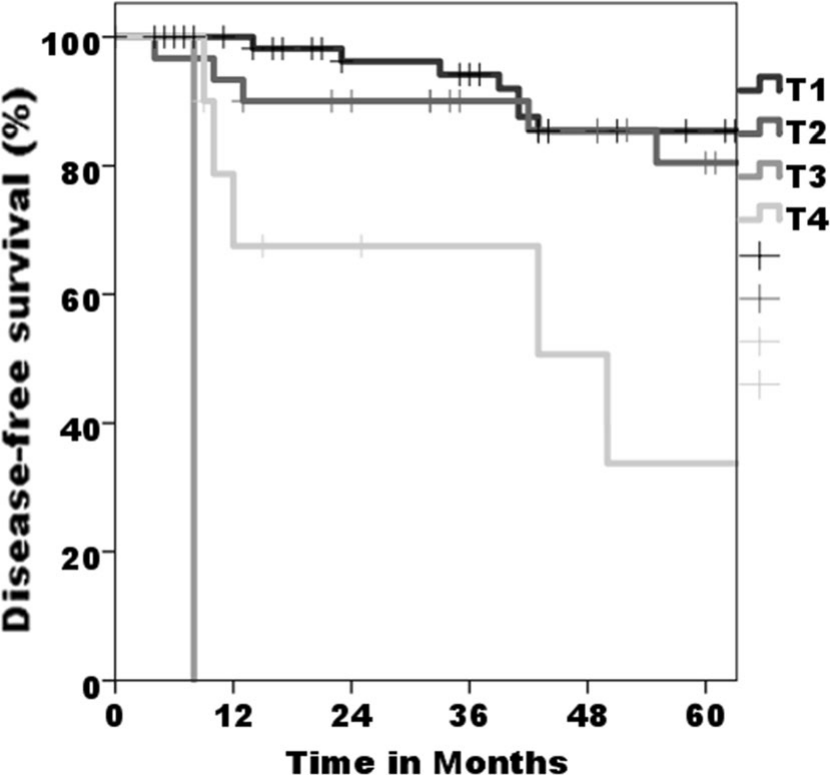
Overall survival based on the tumor size (*p* < 0.001 for *T*4)

In total, 40% (*n* = 42) of the patients developed a relapse (median follow-up interval: 62.3 ± 44.2 months). Second cancer (*n* = 21, 20%) and local recurrence (*n* = 18, 17%) were detected most often as a primary recurrence type. Regional relapse occurrence was rare: isolated in a single case and, an additional two cases also experienced local recurrence (*n* = 2, 2%). On an average, first recurrence took place after 34.4 ± 33.6 months, whereas 26 patients (24.5%) showed one single relapse, sixteen patients (15.1%) developed two or more recurrences; 2% (*n* = 2) showed up to six recurrences. The average of 19.8 ± 23.2 months passed before the second recurrence and 17.9 ± 19.9 months between the second and third recurrence. A noteworthy influence of advanced *T*-status *T*3/*T*4 on presence of tumor relapse in the follow-up could be found (*p* = 0.004, Fig. 1). No influence could be found for presence of lymph node metastasis (*p* = 0.154), grading (*n* = 0.251), or resection margin (*p* = 0.137).

In multivariate analysis, there was no epidemiological factor associated with tumor recurrence (gender, age, tumor localization, positive risk factors, all: *p* > 0.05) of the analyzed cohort (Table 3).

**Table 3:** Presence of Relapse according to different clinical parameters at the point of initial diagnosis

		No relapse	Relapse	Portion of relapse to the respective factor %	
*T*1	*n* = 62	*n* = 40	n = 22	35	*p* = 0.734
*T*2	*n* = 30	*n* = 17	n = 13	43	
*T*3	*n* = 1	*n* = 0	n = 1	100	
*T*4	*n* = 10	*n* = 5	n = 5	50	
*G*1	*n* = 46	*n* = 30	n = 16	35	*p* = 0.444
*G*2	*n* = 54	*n* = 31	N = 23	43	
*G*3	*n* = 3	n = 1	n = 2	67	
*N*0	*n* = 85	56	32	36	*p* = 0.187
*N* +	*n* = 18	8	10	56	
Male	*n* = 46	29	17	38	*p* = 0.094
Female	*n* = 57	33	24	41	
Positive RF	*n* = 67	41	26	41	*p* = 0.715
Negative RF	*n* = 36	22	14	38	
Positive UD	*n* = 73	41	32	45	*p* = 0.075
Negative UD	*n* = 30	23	7	26	
Upper jaw	*n* = 15	7	8	53	*p* = 0.338
Buccal mucosa	*n* = 26	17	9	35	
Tongue	*n* = 30	19	11	37	
Lower jaw	*n* = 23	10	13	57	
Mouth floor	*n* = 12	11	1	8	
Age ≤ 50 year	*n* = 22	17	5	23	*p* = 0.175
Age > 50 year	*n* = 84	47	37	44	

**Fig. 2:**
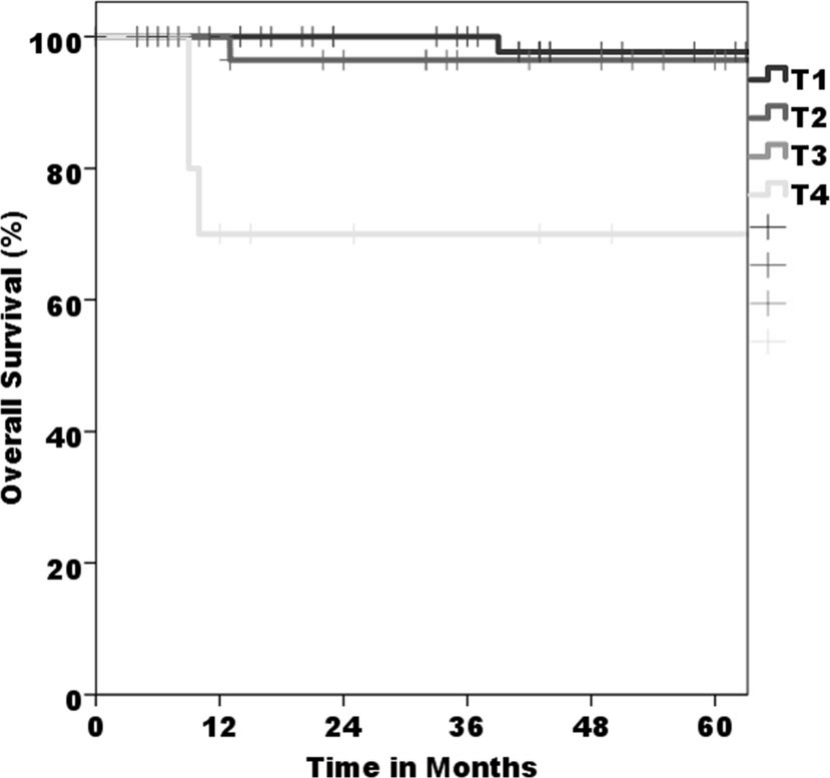
Disease-free survival based on the tumor size (*p* = 0.004 for *T*4)

For the overall survival, within the median follow-up interval of 62.3 ± 44.2 months, fourteen patients (13%) died. There was a significant influence on impaired overall survival for larger tumor size *T*3/*T*4 versus *T*1/*T*2 (*p* < 0.001, Fig. 2), and a noteworthy influence of presence or absence of cervical metastasis (*p* = 0.047) and presence or absence of relapse (*p* = 0.036) in the respective Kaplan–Meier plots and log-rank Mantel–Cox regression. In multivariate analysis, no influence was seen for the tested epidemiological data (gender, age, underlying diseases, tumor localization) or for insufficient resection status (all *p* > 0.05), tumor relapse (*p* = 0.186), and presence of cervical metastasis (*p* = 0.061).

## Discussion

In this retrospective study, a total of 103 patients with OLP-OSCC were re-examined. Of particular interest, there was moderate rates of initial lymph node metastases, as well as an aggressive recurrence pattern, as compared to OSCC.

Apart from classical life-style risk factors like tobacco and alcohol [13, 14], the group of oral premalignant diseases belongs to the most important risk factors responsible for OSCC (3). OLP having a global prevalence of 2% and a malignant transformation rate of 1% is one of the most relevant disorders within oral premalignant diseases [2, 9, 15].

The demographic data collected from our pool of patients with OLP-OSCC differ from the literature when compared to OSCC patients.

Although the average age of presentation (62 ± 14) and a higher average age of female patients are in accordance with existing literature, the male/female ratio in our study is just under 1:1, significantly different from reported literature of 1:3 [16]. In accordance with the literature, we also observed higher rates for men concerning risk factors like parallel consumption of alcohol and tobacco [17]. However, this is an epidemiological distribution often found in patients with OLP [5].

Regarding the anatomic location of OSCC, the provided data in literature are not consistent but depend on the geographical region where the study was performed [16]. Specifically in Asian studies, the OSCC preferably occurred on buccal mucosa, whereas in German-speaking regions, the tongue and the floor of the mouth were the most-affected site [18]. In the present study, the location of the primary tumor was in most cases (> 50%) the tongue and cheek. Notably, the floor of the mouth was affected less frequently. This aberration within the distribution pattern of OLP-OSCC could be attributed to the predilection sites of OLP which specifically manifest symmetrically at the buccal mucosa and tongue [19]. In addition to both these sites, there is a high occurrence of OLP in the gingiva of the maxilla and mandible which could contribute to the high rate (35.9%) of OLP-OSCC in these anatomic regions as compared to OSCC.

The diagnosis was made for most of our patients (~ 90%) at an early stage with a small tumor size (*T*1–2). These findings significantly differ from the numbers stated in the corresponding literature dealing with the classical form of OSCC which describe smaller fractions (~ 1/3 of patients) with *T*1–2 at the time of diagnosis [20]. This early rate of detection in our study could be related to the close follow-up regimen for OLP patients in our center. This close check-up translates to a higher probability of early tumor detection.

At the time of diagnosis, only a minority of the patients (17%) had a positive *N*-status. This number seems higher (29%) in the literature for comparable groups of patients with *T*1–2 tumor size of OSCC [21]. Hence, it is important to keep a close follow-up of OLP patients. In the present analysis, the risk of developing cervical metastasis could clearly be correlated to an increased tumor size and dedifferentiation. The distinct correlation between the rate of metastatic disease and both tumor size and cell dedifferentiation is also reported in the existing literature for OSCC [21, 22]. As patients presented at an early stage at the time of diagnosis, 99% of the patients could receive a curative surgical treatment. Since there were low numbers of patients with an advanced tumor stage (III–IV), adjuvant therapy (radio and/or radio-chemotherapy) had to be conducted exclusively for 24.5% of the patients.

Within the follow-up period, 40% of the patients developed a recurrence, half of them within the first 24 months. Additionally, the first recurrence for OLP-OSCC occurred within a wider time corridor than in OSCC [18, 21, 23–25], and almost half of the patients with recurrence had sequential recurrences with an average of 2.3 recurrences. Here, the time intervals until the next recurrence became shorter with increasing number of recurrences for a patient.

Regarding the location of recurrence according to tumor presentation at the point of initial diagnosis, the lower jaw was affected the most, whereas the floor of the mouth was the least-affected site. Interestingly, when the maxilla was primarily affected, a high proportion of relapses were demonstrated, a pattern that is similar to higher recurrences of OSCC affecting the maxilla.

However, in contrast to OSCC, the tumor size, as well as grade of tumor, and nodal status did not significantly influence the risk of developing recurrences [23, 25, 26]. Interestingly, relapse within the first 5 years led only to a 10% decrease in survival rate, whereas in the corresponding literature of OSCC, relapses had a strong influence on survival in patients with OSCC [21, 24].

In spite of various therapeutic methods available today for treating OSCC, the mortality rates for oral carcinoma in the first 5 years are still high (50%) [16]. However, looking at the analyzed study group, the 5 year survival rate was 93%. It seems that subgroup of OLP-OSCC may show a better overall survival in comparison with OSCC, but tumor recurrence could occur more often and during a longer follow-up period. Mignogna et al*.* [27] found similar outcomes of patients with OLP-OSCC, with a 5 year survival rate of 96.7%. In further agreement with existing literature [20], there is no distinct difference between male and female regarding 5 year survival. Also, the existence of further risk factors had no significant influence on 5 year survival. The evaluation of the cumulative survival showed that an increasing tumor size (*T*3–4) and positive nodal status correlate with poor prognosis, the former being the most significant factor. These observations are identical to those in the literature on clinical factors for OSCC overall survival [20, 21, 28]. Histological grade of dedifferentiation in contrast had no statistically significant effect on cumulative survival. Therefore, the much better survival rates may be attributed to the high rate of identified tumors in an early stage (*T*1–2) as well as to the strict call-recall-system established for patients with OLP-OSCC.

Because of its retrospective manner, this study has some major limitations. Above all, data acquisition depends highly on the accuracy of the clinical records and these may not be sufficient. No information about the time period between first diagnoses of lichen (including different types of lichen) and the date of malignant transformation could be obtained. Furthermore, no definitive conclusion of the possible impact of the type of treatment, the follow-up period and the outcome could be drawn due to lack of specific data.

## Conclusion

The obtained data on OLP-OSCC showed differentiations in epidemiological features, especially gender distribution, in comparison with OSCC. Also, anatomical sites of primary tumor presentation differed from the distribution pattern of classical OSCC, whereas lymph metastasis was not seen more frequently, higher recurrence rates of 40% reflect the aggressive potential of OLP-OSCC in comparison with OSCC. Notably with increasing incidence rate of recurrence, the time interval between two incidences decreased.

In summary, it seems that OPL-OSCC differs significantly in biological behavior in comparison with OSCC. Although the overall prognosis for OLP-OSCC was better than that for OSCC in general, a close check-up for OLP patient is an essential requirement for detection and treatment of a malignant transformation in an early stage.

## Data Availability

All obtained data are analyzed in the study, there are no additional data.
